# The relationship between screen time and attention deficit/hyperactivity disorder in Chinese preschool children under the multichild policy: a cross-sectional survey

**DOI:** 10.1186/s12887-023-04130-x

**Published:** 2023-07-14

**Authors:** Yu Zhou, Xinye Jiang, Ran Wang, Bingbing Guo, Jingfen Cai, Yujing Gu, Jingjing Pei

**Affiliations:** 1 Wuxi Maternity and Child Health Care Hospital, Women’s Hospital of Jiangnan University, Jiangnan University, Wuxi, 214002 China; 2 Department of Neonatology, Children’s Hospital of Fudan University, National Children’s Medical Center, Shanghai, China

**Keywords:** Screen time, ADHD, Preschool children, Multichild families

## Abstract

**Background:**

Long screen time has become a public health problem that cannot be ignored. The association between screen time and attention-deficit/hyperactivity disorder (ADHD) in preschool children has received widespread attention.

**Methods:**

A questionnaire was used to survey 2452 people. ADHD symptoms were assessed by the Conners Child Behavior Scale. Considering that the ADHD symptoms of boys and girls might be different, we stratified the data by gender. Logistic regression model was used for regression analysis. To exclude the influence of multichild family and obesity level, we also conducted a sensitivity analysis. *P* values were two-tailed with a significance level at 0.05.

**Results:**

The results showed that the association between screen time and ADHD symptoms in preschool children was significant (*OR* = 1.826, 95%*CI*: 1.032, 3.232). After grouping the genders, the correlation was not significant. There was an association between screen time and ADHD symptoms in children from families with multiple children. However, after excluding overweight and obese children from the overall population, the association between screen time and ADHD symptoms did not have statistical significance.

**Conclusions:**

The issue of screen time for preschoolers needs to be taken seriously. Although the results indicate a significant correlation between screen time and ADHD symptoms, clearer evidence is needed to provide recommendations to policy makers.

**Supplementary Information:**

The online version contains supplementary material available at 10.1186/s12887-023-04130-x.

## Background

Attention deficit/hyperactivity disorder (ADHD) is one of the most common psychiatric disorders with an estimated worldwide prevalence of 7.2% among children [1]. Children with ADHD have an increased risk of experiencing serious, lifelong impairments in multiple domains of daily functioning [2]. For example, their academic performance may be affected, and they are more likely to be bullied by their peers and ignored by others because of their special behavior in school [3]. Additionally, they may experience more family conflicts [1, 4]. Findings indicate that the development of ADHD symptoms usually starts before school age. Behavioral problems among some children at early stages of development are not temporary [5] and increase the risk of adverse developmental outcomes. Without intervention, ADHD symptoms tend to widen developmental gaps over time [6]. This emphasizes the importance of early detection and intervention.

Increasing attention has been focused on the relationship between screen time and ADHD symptoms. Studies on school-age children [7] and adolescents [8] have shown that screen time is significantly related to attention problems. The association between screen time and ADHD has received widespread attention in preschool children as well. It is estimated that preschool children are exposed to an average of two hours of screen time per day in Canada [9]. Excessive screen time (defined as more than 2.8 h/day) is prevalent in preschool children in Shanghai city (China) [10]. Similar situations exist in other countries and regions. As an integrated part of children’s lives, long screen time has become a public health problem that cannot be ignored. As for the association between screen time and ADHD, two hypotheses have been suggested. First, fast-paced media forces children to repeatedly shift their attention, which increases arousal [11]. With frequent exposure, the reduced arousal level may ultimately lead to ADHD-related behaviors. A second hypothesis [12] argues that the fast pacing of media prevents children from developing attentional focusing skills. Therefore, it is important to clarify the relationship between screen time and ADHD symptoms in preschool children.

In addition, ADHD is more common among boys. Gender may influence the association between screen exposure and ADHD symptoms. The meta-analysis of Nikkelen et al. [12] and their empirical work [13] suggested that boys were more susceptible to the effects of media on ADHD-related behaviors than girls [14]. However, Ansari and Crosnoe [14] found an opposite trend: higher levels of television viewing were related to higher levels of hyperactivity among girls but not among boys. As a result, we also considered the impact of gender in this study. In China, fertility policies are constantly changing. After the implementation of the one-child policy for more than 30 years, the universal two-child policy was implemented in 2015 [15]. By 2021, the three-child policy was implemented [16]. There is a lack of research on screen behavior in multichild families. Researches [17] showed that children have sibling spend more time using screens, which may be related to the dispersed parenting energy of parents. In our study, we also considered whether preschoolers who had younger siblings at home had more difficulties because of poor parenting.

## Methods

In October 2019, we conducted stratified sampling to randomly select a kindergarten with more than 200 people from 6 districts and 2 county-level city in Wuxi City. We conducted a survey on all children in the kindergarten in 8 community kindergartens. A total of 2560 questionnaires were distributed. The questionnaire was completed by the child’s main caregivers. A total of 2452 questionnaires were returned (response rate 95.8%), including 1348 (55.0%) boys and 1104 girls (45.0%). Informed consent was obtained before filling in the questionnaire. This study was reviewed by the Ethics Committee of Wuxi Maternal and Child Health Hospital. The study was carried out in accordance with the Declaration of Helsinki.

Trained members of the research group served as investigators. We informed parents of the purpose and methods of the study and issued a questionnaire after obtaining informed consent. The investigator’s phone number was marked at the beginning and end of the questionnaire. Parents could contact researchers over the phone if they encountered any problems during the completion process. Questionnaire information included general demographic characteristics, the mother’s pregnancy and childbirth status, number of children, ranking of children, screen time, and Conners Child Behavior Scale data. When sorting the data, we investigated any unclear answers in the questionnaire. This process was conducted by the child’s teacher.

The height and weight data were measured by professional health teachers. The corresponding body mass index was calculated using the following formula: body mass index (BMI) = weight (kg)/height² (m²). Obesity was assessed by linking body mass index with the adult cutoff point [18].

### Assessment of screen time

Parents were asked to recall the children’s daily screen time (including watching TV, playing mobile phones, playing on tablets/computers, and using game consoles) in the past six months. The screen time was divided into weekdays and on weekends. The description of screen time in the questionnaire was as follows: How many hours does your child spend on screen (watching TV/playing computer/mobile phone/tablet) every day (weekdays/weekends)? In the questionnaire, we asked about the screen time on weekdays and weekends separately. We then calculated the average screen time for our sample population. The average screen time was calculated as (Screen time on weekdays*five + Screen time on weekend*two)/seven [19]. According to the Implementation Plan of the Prevention and Control of Children and Adolescents’Myopia [20],we consider daily screen time exceeding 1 h as excessive screen time.

### Assessment of ADHD symptoms

We used the Conners Child Behavior Scale to assess ADHD symptoms in children. The Conners Children’s Behavior Scale was used to assess the behavior problems of children aged 3–16 years in China and has been proven to be valid and reliable [21]. This study used a parent questionnaire of 48 items, with each item scored according to a four-level scoring method. This questionnaire included six dimensions, including character problems, learning problems, psychosomatic problems, hyperactivity/impulsiveness, anxiety, and hyperactivity index. The higher the score, the more serious the behavior problem. According to the literature, we consider hyperactivity index greater than 1.5 as the presence of ADHD symptoms [22].

We used Epidata 3.1 software to enter the data. Microsoft Excel (2016) was used to generate figures. All analyses were conducted in SPSS software. Frequencies and percentages were summarized for categorical variables. For continuous variables, mean and standard deviation (SD) were calculated. The chi-square test and *t* test were used to test the distribution differences. Considering that the hyperactivity index of boys and girls might be quite different, this study stratified all data by gender. Logistic regression was used for regression analysis. To exclude the influence of overweight and obese children, we also conducted a sensitivity analysis. For the convenience of explanation, we named the model that included the screen time on weekdays and weekends as model 1, and the model that included the average screen time as model 2. The odd ratio (*OR)* value and 95% confidence intervals (95% *CI*) were reported. *P* values were two-tailed with a significance level at 0.05.

## Results

Basic information about the participants is shown in Table 1. The average age of the participants was 5.25 ± 0.63 years. 1348 boys (55.0%) participated in the survey. 217 (8.8%) of participants were overweight or obese. A total of 50.8% of participants were the only child in the family. 457 (19.4%) children had screen time > 1 h on weekdays, 1130 (48.0%) children had screen time > 1 h on weekends, and 967 (39.4%) children had average screen time > 1 h per day. A total of 20.9% of boys had more than one hour of screen time on weekdays, which was higher than the proportion of girls (*P* = 0.040). The proportion of screen time exceeding one hour on weekend was nearly half for all subjects, with boys being higher (50.3% for boys and 45.3% for girls, *P* = 0.015). The proportion of boys who spent more than 1 h on the screen was higher than that of girls in average screen time (44.7% for boys and 37.6% for girls, *P <* 0.001). A total of 3.5% of preschool children with ADHD symptoms was identified by this survey.

**Table 1:** Demographic characteristics among participants between different genders

Group		Total	Boys	Girls	*P*
n (%)		2452 (100.0)	1348 (55.0)	1104 (45.0)	
Children age (years) (Mean ± SD)		5.25 ± 0.63	5.24 ± 0.64	5.25 ± 0.62	0.856
Age at pregnancy (years) (Mean ± SD)		27.70 ± 4.01	27.64 ± 4.00	27.77 ± 4.02	0.422
Premature, n (%)					0.001
	Non-premature	2337 (95.3)	1220 (54.0)	59 (72.0)	
	Premature delivery	115 (4.7)	1040 (46.0)	23 (28.0)	
Paternal education levels, n (%)					0.079
	Junior high school and below	205 (8.4)	128 (9.5)	77 (7.0)	
	Senior high school	356 (14.5)	191 (14.2)	165 (14.9)	
	College degree and above	1766 (72.0)	954 (70.8)	812 (73.6)	
	Unknown	125 (5.1)	75 (5.6)	50 (4.5)	
Maternal education levels, n (%)					0.222
	Junior high school and below	266 (10.9)	154 (11.4)	112 (10.1)	
	Senior high school	320 (13.1)	180 (13.4)	140 (12.7)	
	College degree and above	1762 (71.9)	949 (70.4)	813 (73.6)	
	Unknown	104 (4.2)	65 (4.8)	39 (3.5)	
Number of children in family, n (%)					0.106
	One	1236 (50.8)	697 (52.2)	539 (49.0)	
	Two and more	1199 (49.2)	637 (47.8)	562 (51.0)	
Overweight or obesity, n (%)					< 0.001
	Yes	217 (8.8)	142 (10.5)	75 (6.8)	
	No	2235 (91.2)	1206 (89.5)	1029 (93.2)	
Screen time > 1 h, n (%)					
	Weekdays	457 (19.4)	270 (20.9)	187 (17.5)	0.040
	Weekend	1130 (48.0)	647 (50.3)	483 (45.3)	0.015
	Average	967 (39.4)	568 (44.7)	399 (37.6)	< 0.001
ADHD symptoms, n (%)		82 (3.5)	59 (4.6)	23 (2.2)	< 0.001

Note:The chi-square test (categorical variables) and t test (continuous variables) were used to test the distribution difference between boys and girls. The *P*-value is the statistical significance of the difference test

The distribution difference of children with or without ADHD symptoms in each factor was shown in Table 2. Compared to children without ADHD symptoms, children with ADHD symptoms have a higher proportion of exposure to screens > 1 h, whether on weekdays (*P <* 0.001) or weekends (*P* = 0.045). Boys had a higher proportion of ADHD symptoms (*P <* 0.001).

**Table 2:** Distribution characteristics of ADHD symptoms among preschool children Note:The chi-square test (categorical variables) and t test (continuous variables) were used to test the distribution difference

Group		Non-ADHD symptoms	ADHD symptoms	*P*
Total, n (%)		2260 (96.5)	82 (3.5)	
Screen time > 1 h in weekdays, n (%)				0.001
	No	1800 (81.6)	54 (66.7)	
	Yes	407 (18.4)	27 (33.3)	
Screen time > 1 h in weekend, n (%)				0.045
	No	1157 (52.7)	34 (41.5)	
	Yes	1038 (47.3)	48 (58.5)	
Average screen time > 1 h, n (%)				0.003
	No	1297 (59.5)	35 (43.2)	
	Yes	884 (40.5)	46 (56.8)	
Children age (years) (Mean ± SD)				0.060
		5.24 ± 0.63	5.11 ± 0.67	
Gender, n (%)				0.001
	Boys	1220 (54.0)	59 ( 72.0)	
	Girls	1040 (46.0)	23 (28.0)	
Age at pregnancy (years) (Mean ± SD)				0.104
		27.71 ± 3.95	26.99 ± 4.00	
Premature, n (%)				0.922
	Non-premature	2155 (95.4)	78 (95.1)	
	Premature delivery	105 (4.6)	4 (4.9)	
Paternal education levels, n (%)				0.030
	Junior high school and below	163 (7.3)	13 (15.9)	
	Senior high school	323 (14.3)	13 (15.9)	
	College degree and above	1657 (73.3)	52 (63.4)	
	Unknown	116 (5.1)	4 (4.9)	
Maternal education levels, n (%)				0.043
	Junior high school and below	213 (9.4)	15 (18.3)	
	Senior high school	292 (12.9)	12 (14.6)	
	College degree and above	1664 (73.6)	51 (62.2)	
	Unknown	91 (4.0)	4 (4.9)	
Number of children in family, n (%)				0.628
	One	1145 (50.9)	44 (53.7)	
	Two and more	1103 (49.1)	38 (46.3)	
Overweight or obesity, n (%)				0.095
	No	1749 (77.4)	57 (69.5)	
	Yes	511 (22.6)	25 (30.5)	

Note:The chi-square test (categorical variables) and t test (continuous variables) were used to test the distribution difference.

After adjusting for control variables, logistic regression model showed a significant association between weekdays screen time > 1 h and ADHD symptoms among preschool children (*OR* = 1.826, 95%*CI*: 1.032, 3.232) (Table 3). However, weekend exposure was not associated with ADHD symptoms. As age increases, the likelihood of preschool children suffering from ADHD symptoms decreases (*OR* = 0.701, 95%*CI*: 0.495, 0.992). Compared to boys, girls were less likely to have ADHD symptoms (*OR* = 0.494, 95%*CI*: 0.301, 0.812). When conducted a subgroup analysis by gender, we did not find an association between screen time > 1 h and ADHD symptoms. After averaging the total screen time for each day, the association between screen time and ADHD symptoms persisted for all subjects (Table 4). However, the results of subgroup analysis are not consistent with the overall population. In the sensitivity analysis, no differences were found in the main conclusions of children in families with multiple children (Supplementary Table 1). There was a significant correlation between screen time and ADHD symptoms of children in families with multiple children. The correlation was not significant after subgroup analysis. We additionally tested the children except for those who were overweight and obese (Supplementary Table 2). We did not find the association between screen time and ADHD symptoms in those children.

**Table 3:** Logistic regression results of ADHD symptoms among preschool children

Group		Total *OR* (95%*CI*)	Subgroup analysis	
			Boys *OR* (95%*CI*)	Girls *OR* (95%*CI*)
Screen time > 1 h				
	Weekdays	**1.826 (1.032, 3.232)**	1.723 (0.861, 3.448)	2.270 (0.814, 6.332)
	Weekend	1.086 (0.635, 1.858)	0.951 (0.505, 1.789)	1.504 (0.537, 4.210)
Children age (years)		**0.701 (0.495, 0.992)**	0.786 (0.520, 1.188)	0.522 (0.265, 1.030)
Gender				
	Boys	Ref.	—	—
	Girls	**0.494 (0.301, 0.812)**	—	—
Age at pregnancy (years)		0.990 (0.926, 1.058)	0.986 (0.910, 1.068)	1.004 (0.890, 1.134)
Premature				
	Non-premature	Ref.	Ref.	Ref.
	Premature delivery	1.023 (0.361, 2.896)	0.659 (0.155, 2.802)	2.112 (0.435, 10.263)
Paternal education levels				
	Junior high school and below	Ref.	Ref.	Ref.
	Senior high school	2.009 (0.503, 8.016)	1.564 (0.316, 7.730)	2.429 (0.176, 33.602)
	College degree and above	1.312 (0.345, 4.991)	0.729 (0.147, 3.609)	2.303 (0.205, 25.849)
	Unknown	1.352 (0.384, 4.761)	1.037 (0.244, 4.406)	1.639 (0.147, 18.291)
Maternal education levels				
	Junior high school and below	Ref.	Ref.	Ref.
	Senior high school	0.691 (0.275, 1.733)	1.030 (0.322, 3.291)	0.339 (0.069, 1.674)
	College degree and above	0.608 (0.248, 1.487)	0.840 (0.268, 2.629)	0.295 (0.067, 1.300)
	Unknown	1.087 (0.275, 4.299)	0.739 (0.115, 4.744)	1.657 (0.231, 11.895)
Number of children in family				
	One	Ref.	Ref.	Ref.
	Two and more	0.875 (0.540, 1.416)	1.166 (0.657, 2.066)	0.437 (0.173, 1.100)
Overweight or obesity				
	No	Ref.	Ref.	Ref.
	Yes	1.287 (0.782, 2.119)	1.481 (0.838, 2.617)	0.817 (0.264, 2.524)

Note: Some numbers in bold meant that the statistical significance of *OR* existed

**Table 4:** The relationship between average screen time and ADHD symptoms of preschool children

Group		Total *OR* (95%*CI*)	Subgroup analysis	
			Boys *OR* (95%*CI*)	Girls *OR* (95%*CI*)
Model 1				
Screen time > 1 h				
	Weekdays	**1.826 (1.032, 3.232)**	1.723 (0.861, 3.448)	2.270 (0.814, 6.332)
	Weekend	1.086 (0.635, 1.858)	0.951 (0.505, 1.789)	1.504 (0.537, 4.210)
Model 2				
Screen time > 1 h				
	Average	**1.615 (1.008, 2.586)**	1.420 (0.814, 2.479)	2.249 (0.926, 5.464)

Note: Some numbers in bold meant that the statistical significance of *OR* existed. Average screen time > 1 h was obtained by integrating screen time on weekdays and weekends; The regression equation of model 1 included the screen time on weekdays and weekends; The regression equation of model 2 included the average screen time. Both models adjusted for child age, sex (unadjusted in subgroup analysis), maternal age at gestation, premature, parental education level, number of children in family, overweight and obesity

## Discussion

This study demonstrated the association between screen time and ADHD symptoms in preschool children. Gender might affect the association between screen time and ADHD symptoms seriously. This study found that there is an association between screen time and ADHD symptoms in children from families with multiple children. However, after excluding overweight and obese children from the overall population, the association between screen time and ADHD symptoms did not have statistical significance. The results of these subgroup analyses might suggest that the correlation between screen time and ADHD symptoms may be influenced by other factors, such as gender and obesity level.

Electronic devices are deeply involved in children’s lives. In our study, 457 (19.4%) children had screen time > 1 h on weekdays, 1130 (48.0%) children had screen time > 1 h on weekends. Overexposure to screens in early childhood has become a common phenomenon. Some studies showed that the proportion of children who use electronic products per day more than 2 h a day is 60% in 40 countries in Europe and North America [23, 24] and 57.3% in China [25]. Both the World Health Organization and China recommend that preschool children use media for less than 1 h per day to help young children use media devices appropriately [26]. However, extensive exposure to electronic screens in early childhood can comprehensively affect children’s health and functions. Prolonged screen-time may displace time spent in other activities such as play out in the open. This “all day counts” approach puts each behavior on a continuum, where a decrease in one leads to an increase in the other. Moreover, researches had shown that the screen time tends to stabilize in early childhood [27]. That is to say that the intervention should be carried out on their video behavior from early childhood to decrease the screen time and achieve greater health benefits.

In our research, the relationship between screen time and ADHD symptoms was statistically significant (*OR* = 1.826, 95%*CI*: 1.032, 3.232). Other studies had also confirmed this conclusion. Nikkelen et al. [11] and Ferguson [28] found statistically small but significant pooled correlations between screen media use and ADHD-related behaviors. Other studies have shown that the correlation between screen time and ADHD symptoms is not significant [29]. Even if there is a correlation, the coefficient of correlation may be small. When we conducted subgroup analysis by gender, there was no statistically significant association between screen time and ADHD symptoms. This might be due to the excessive influence of gender on ADHD symptoms. A better research design needs to be implemented in future studies to determine the overall impact of screen time on children’s health. In the results, we found that the younger the age, the higher the likelihood of having ADHD symptoms. This may be related to the assessment of ADHD symptoms is a questionnaire filled out by parents. At this stage, parents have high expectations for their children’s attention, which is not match the development of children’s attention. The mechanism of screen time on hyperactivity symptoms is unclear. It may be attributed to repetitive attentional shifts and multitasking, which can impair executive functioning [30]. The rapidly changing focus of most programming in electronic media may impair children’s ability to concentrate [12]. When children spend more time on electronic screens, they have less time for offline social and physical activities, which further reduces their cognitive function and mental health. Regardless of the correlation mechanism between them, it is a worthwhile topic to regulate screen behaviors in preschool children.

In our study, we found a significant correlation between screen time and ADHD symptoms in families with multiple children. This is consistent with our original hypothesis. After China abolished the one-child policy, many families now have several children [15, 16]. In our study, half of households had multiple children. When children enter kindergarten, many families choose to have one or more additional children. Parents may spend more energy caring for children and may be more likely to use electronics to comfort their children [31], especially when children are at home on weekends and away from their teachers and peers. Unfortunately, this result could only serve as an exploration of the relationship. More rigorous design and analysis are needed to examine the correlation between ADHD symptoms and screen time in families with multiple children.

In addition, the association between screen time and ADHD symptoms was not significant in preschool children except for those who were overweight and obese in our study. The result also indirectly reveals that obesity levels may affect the correlation between ADHD symptoms and screen time [32]. Screen time can increase sedentary time, which can affect BMI. In turn, obese children may also be more inclined to use electronic products for long periods for physical reasons [33]. This suggests that further analysis need to control obesity, gender and other factors in the design stage.

### Limitations

There are some limitations to this study. First, this was a self-reported questionnaire and may be subject to information bias. In addition, we did not consider the presence or absence of parent‒child companionship during screen time or the video content [34, 35]. All of these factors might lead to changes in the effect of screen time on children’s behavior. Thirdly, the study did not consider the screen time of preschool children at home during winter and summer vacations. Finally, the cross-sectional data used in these analyses prevent making inferences between screen time and ADHD symptoms over time.

## Conclusion

In conclusion, this study confirmed the association between screen time and ADHD symptoms in preschool children. The results of gender and obesity levels might indicate that the association may be influenced by multiple factors. Therefore, when bring forth a corresponding proposal to the public, we should be more cautious. Taken together, the results of this study suggest that early childhood could be the critical juncture at which to prioritize preventive interventions focusing on problematic screen time.

## Electronic supplementary material

Below is the link to the electronic supplementary material.

Supplementary Material 1

## Data Availability

The data underlying this article cannot be shared publicly due to the privacy of individuals who participated in the study. The data will be shared on reasonable request to the corresponding author.

## Abbreviations

ADHD: Attention-deficit/hyperactivity disorder
