# Supplementary material for: The relationship between screen time and attention deficit/hyperactivity disorder in Chinese preschool children under the multichild policy: a cross-sectional survey
Source: BMC Pediatr. 2023 Jul 14;23:361. doi: 10.1186/s12887-023-04130-x (PMC10347872; doi:10.1186/s12887-023-04130-x)
Supplement: Supplementary file 1 — Supplementary Material 1 [file 12887_2023_4130_MOESM1_ESM.docx]

| Supplementary table 1 The relationship between screen time and ADHD symptoms in preschool children in multi-child families | | | | |
| --- | --- | --- | --- | --- |
| Group | | Total  OR (95%CI) | Subgroup analysis | |
|  |  |  | Boys  OR (95%CI) | Girls  OR (95%CI) |
| Model 1 | |  |  |  |
| Screen time > 1 hour | |  |  |  |
|  | Weekdays | **2.443 (1.097, 5.441)** | 2.354 (0.890, 6.229) | 3.142 (0.742, 13.302) |
|  | Weekend | 1.214 (0.535, 2.752) | 0.832 (0.328, 2.108) | 5.407 (0.586, 49.863) |
| Model 2 | |  |  |  |
| Screen time > 1 hour | |  |  |  |
|  | Average | **2.115 (1.035, 4.321)** | 1.644 (0.732, 0.692) | 4.816 (0.941, 24.653) |

Note: Some numbers in bold meant that the statistical significance of *OR* existed. Average screen time > 1 hour was obtained by integrating screen time on weekdays and weekends; The regression equation of model 1 included the screen time on weekdays and weekends; The regression equation of model 2 included the average screen time. Both models adjusted for child age, sex (unadjusted in subgroup analysis), maternal age at gestation, premature, parental education level, number of children in family, overweight and obesity.

| Supplementary table 2 The relationship between screen time and ADHD symptoms in preschool children except for those who were overweight and obese | | | | |
| --- | --- | --- | --- | --- |
| Group | | Total  OR (95%CI) | Subgroup analysis | |
|  |  |  | Boys  OR (95%CI) | Girls  OR (95%CI) |
| Model 1 | |  |  |  |
| Screen time > 1 hour | |  |  |  |
|  | Weekdays | 1.814 (0.886, 3.714) | 1.719 (0.674, 4.384) | 2.346 (0.724, 7.600) |
|  | Weekend | 0.863 (0.447, 1.665) | 0.653 (0.286, 1.492) | 1.377 (0.434, 4.363) |
| Model 2 | |  |  |  |
| Screen time > 1 hour | |  |  |  |
|  | Average | 1.160 (0.662, 2.031) | 0.887 (0.442, 1.783) | 1.985 (0.743, 5.302) |

Note: Some numbers in bold meant that the statistical significance of *OR* existed. Average screen time > 1 hour was obtained by integrating screen time on weekdays and weekends; The regression equation of model 1 included the screen time on weekdays and weekends; The regression equation of model 2 included the average screen time. Both models adjusted for child age, sex (unadjusted in subgroup analysis), maternal age at gestation, premature, parental education level, number of children in family, overweight and obesity.
